# Depression Following COVID-19 Lockdown in Severely, Moderately, and Mildly Impacted Areas in China

**DOI:** 10.3389/fpsyt.2021.596872

**Published:** 2021-02-19

**Authors:** Xuerui Han, Shuquan Chen, Kaiwen Bi, Zeyun Yang, Pei Sun

**Affiliations:** ^1^Department of Clinical and Counseling Psychology, Teachers College, Columbia University, New York, NY, United States; ^2^Department of Psychology, Columbia University, New York, NY, United States; ^3^Department of Psychology, School of Social Science, Tsinghua University, Beijing, China

**Keywords:** exposure, stress, lockdown, COVID-19, depression

## Abstract

**Background:** The Coronavirus Disease 2019 (COVID-19) pandemic has led to overwhelming levels of distress as it spread rapidly from Wuhan, Hubei province to other regions in China. To contain the transmission of COVID-19, China has executed strict lockdown and quarantine policies, particularly in provinces with the highest severity (i.e., Hubei). Although the challenges faced by individuals across provinces may share some similarities, it remains unknown as to whether and how the severity of COVID-19 is related to elevation in depression.

**Methods:** The present study compared depression among individuals who lived in mildly, moderately, and severely impacted provinces in China following the lockdown (*N* = 1,200) to norm data obtained from a representative sample within the same provinces in 2016 (*N* = 950), and examined demographic correlates of depression in 2020.

**Results:** Residents in 2020, particularly those living in more heavily impacted provinces, reported increased levels of depression than the 2016 sample. Subsequent analyses of sub-dimensions of depression replicated the findings for depressed mood but not for positive affect, as the latter only declined among residents in the most severely impacted area. Increased depressed mood was associated with female, younger age, fewer years of education, and being furloughed from work, whereas reduced positive affect was associated with younger age and fewer years of education only.

**Conclusions:** This study underscored the impact of COVID-19 on depression and suggested individual characteristics that may warrant attention.

## Introduction

In December 2019, an unknown and infectious disease broke out in Wuhan, China, which was declared a pandemic by the World Health Organization (1) and officially referred to as the Coronavirus Disease 2019 (COVID-19) on February 11th, 2020. Since then, it has taken a tremendous toll on individuals, families, and communities, infecting and claiming millions of lives worldwide (2). In addition to adapting to a new reality dominated by fear of viral contagion as well as social isolation due to lockdown and quarantine, individuals have experienced devastating losses in multiple domains of life. The long-term effects may extend further than the duration of the lockdowns. As individuals start to seek reemployment and a return to normal life, depressive symptoms may emerge as feelings of sadness and loss grow beyond fear and anxiety, when they realize that normality continues to remain elusive. The present study aims to compare depressive symptoms among Chinese residents in provinces that were severely, moderately, mildly hit by the pandemic in early May of 2020 with pre-pandemic norm and identify demographic correlates of depression after the lockdown.

### Elevated Psychological Distress Amidst the Outbreak of COVID-19

COVID-19 is particularly disruptive as it imposes widespread and severe restrictions without a certain end date, presents a complex combination of stressful life events, and blocks access to protective factors (3).

Fear of contracting the virus is probably one unique stressor that the COVID-19 has imposed on individuals. Over one-third of respondents expressed increased concern and excessive anxiety about viral infection, even when the risk was estimated to be low in early 2020 (4). Loss due to COVID-19 can range in severity and duration, be direct (i.e., infection) or indirect [i.e., child mortality in low-income countries; Roberton et al. (5)], present-oriented (i.e., unemployment), or future-oriented (i.e., uncertainty of academic progression) and on the individual (i.e., increased psychological distress), or the collective (i.e., an overstretched medical system) level, resulting in varying elevation in psychological distress. Another ramification of COVID-19 is an increase in the feelings of disgust toward outgroups that are believed to pose an elevated risk of infection (6). Disease avoidance arises from people's evolutionary tendency to maintain health (7), but it unfortunately contributes to prejudice against national subgroups (i.e., the residents of Hubei Province, China). Hubei residents encountered increasing social exclusion and stigmatization in forms of in-person verbal assault, destruction of property, being denied employment opportunities or access to public facilities and a general violation of fairness (8). Given that the adverse impact of discrimination on people's mental health has been well-documented (9), it is possible that levels of depression in Hubei residents might be further aggravated by such experiences.

### Depression Following the Lockdown

COVID-19 led to unprecedented policies of quarantine in an attempt to contain the pandemic, starting with Wuhan in Hubei Province. Enforced by government and community officials, stringent lockdown measures prohibited residents from leaving the city, restricted each household to send one person to purchase groceries twice a week, and banned the private use of cars (10). To further limit group activities, the local government also took steps to reward individuals who reported neighbors breaking social distancing rules (10). Inevitably, mandatory quarantine generated common challenges such as working from home while balancing childcare, experiencing wage loss, and lacking food supplies, and clashed with the fundamental human need for connection and belonging (11). Forced social isolation reduced social and physical contacts with others, thereby generating elevated depressed mood, emotional disturbance, boredom, frustration, and blocking access to effective coping strategies such as seeking social support (12, 13).

### Stress Exposure: Severely, Moderately, and Mildly Impacted Areas

In the current literature, a number of studies have investigated the association between combat exposure and the prevalence of PTSD and mood disorders, provided that exposure to trauma is positively related to the severity of symptoms (14–16). Other studies have followed to suggest that the magnitude of exposure to a variety of adverse events, including natural disasters (17), childhood maltreatment (18), and racism (9) is associated with subsequent depressive symptoms and overall maladjustment (17). Studies on different epidemics, including COVID-19, the severe acute respiratory syndrome (SARS), and Middle East respiratory syndrome (MERS), reached same conclusions that the level of disease exposure was a substantial risk factor for developing psychological problems (19). Specifically, health care workers or employees in high-exposure-risk locations (i.e., Wuhan vs. other cities in Hubei province vs. outside Hubei province) were significantly more likely to exhibit symptoms of depression, anxiety, distress, insomnia (20), and PTSD symptoms (21). Therefore, it is reasonable to suspect that different patterns of mental health issues existed across provinces that were exposed to COVID-19 to different degrees.

One way to determine the severity of exposure for each province is calculating the infection rate. Specifically, the provincial number of COVID-19 cases was divided by the total number of regular residents (in millions) for each province, using the government census data. The infection rate, ranging from 2.99 to 1,151.41, was utilized as an index to compare severity and categorize all regions into mildly impacted (MiA, i.e., 2.99–9.03), moderately impacted (MoA, i.e., 12.24–27.53), and severely impacted areas (SeA, 1,151.41). By May 10th, 2020, Hubei province is considered the highest in severity with an infection index of 1,151.41 to represent SeA. For MoA, Guangdong and Zhejiang provinces were chosen, with 22.10 and 14.00 per million residents contracting the virus. Shanxi and Sichuan represented MiA, since both provinces had lower infection indexes of 5.33 and 6.73, respectively. Hubei, Guangdong, Zhejiang, Sichuan, and Shanxi provinces are geographically proximate.

### Demographics Correlates of Depression Following the Lockdown

Recent research has identified potential correlates such as younger age, being single, fewer years of education, female gender, student status, pre-existing physical symptoms, and poor perceived general health (22–24). In an attempt to replicate previous findings and generate novel explanations, we included not only gender, age, years of education attained, and marital status, but also annual income and changes in work or wage resulting from disruptions caused by COVID-19 into the analysis. To our knowledge, these two factors were rarely discussed in combination. Early works showed that unemployment and economic insecurity had detrimental effects on one's self-rated health and psychological health both short-term and long-term (25–27). Following the Great Economic Regression from 2007 to 2009, recession-related stressors such as increased debt, reduced budget, unemployment, and inability to pay rent, were associated with higher odds of developing depression, generalized anxiety, panic, substance use even years later (28). Similarly, Wilson et al. (29) have found that increased job insecurity and financial concerns were differentially associated with heightened depressive and anxious symptoms. Brooks et al. (12) have proposed that individuals with lower annual income prior to the pandemic might be more affected by financial uncertainties and require additional support than those with higher income. Taking both income level and change in employment status into account, we intended to investigate which factor was more strongly related to increased depression in face of COVID-19. The majority of research on COVID-19 has used univariate analyses to explore these relationships, whereas our study conducted multivariate analyses with a forward stepwise procedure (30), which could provide information on the significance of the relationships and the size of the effects as well as the structure and the interaction effect of multiple covariates while adjusting for potential confounding factors (31).

### The Current Investigation

The current investigation aims to compare depression along with its two subdimensions, measured by the Center for Epidemiological Studies – Depression (CES-D) scale among residents living in mildly, moderately, and severely impacted provinces in 2020 and norm data in 2016. In addition, we investigated how potential demographic factors relate to depression. Taken together, we proposed the following hypotheses: (1) Residents in 2020 would exhibit greater levels of depression than residents in 2016. (2) Among residents surveyed in 2020, those living in SeA would exhibit the highest levels of depression, followed by those living in MoA, followed by those living in MiA. (3) Following the lockdown, those who were female, furloughed, or achieved lower levels of education and income would exhibit greater levels of depression than their counterparts. The other demographic factors examined were exploratory in nature, including age (i.e., while age was often perceived to be negatively correlated with depression, the elderly might have suffered the most during the pandemic) and marital status (i.e., although this factor was often discussed, research results were inconsistent).

## Methods

### Participants and Data

To capture levels of depression prior to COVID-19, depression norm data collected in 2016 from the China Family Panel Studies [CFPS; (32)], a nationally representative survey of Chinese communities, families, and individuals, was obtained. CFPS is conducted by the Institute of Social Science Survey (32) of Peking University, China, attempting to provide a comprehensive overview of the citizen's health, mental well-being, educational attainment, family income, parental practices, social relationships, and others. CFPS collects data every 2 years, and the most recent data set was sampled in 2018. Nonetheless, it did not measure the 20-item CES-D scale and could not be used. Instead, the openly available 2016 data set included the CES-D 20-item scale and served as the baseline depression norm, which is comparable to those of two other Chinese studies (33, 34). Eligibility criteria included age between 18 and 65, responding to all 20 items on the CES-D scale, and living in Hubei, Guangdong, Zhejiang, Sichuan, or Shanxi at the time when the survey was taken. Data were collected in person, through the phone, or using the internet. Participants who did not comply with data collection (e.g., invariance of response or non-compliance) were excluded.

All participants in the 2020 sample were recruited simultaneously from May 10 to 20, 2020, adopting the same eligibility criteria. The study was launched using the Questionnaire Star, a Chinese survey platform that facilitates high-quality data collection. A link to the survey was disseminated via popular social media platforms such as Wechat, Weibo, and Zhihu. Participation was voluntary and anonymous. Respondents were debriefed about the nature and aim of the study and gave informed consent. This study was approved by the Institutional Review Broad (IRB) at the Department of Psychology, XXX (Masked for blind review) University.

### Measures

The survey consisted of demographic information and depression scores. Participants were asked to report their age, gender, education level, marital status, their annual individual income prior to COVID-19 and changes in their employment and income status (i.e., “Decreased income,” “No change in income,” “Increased income,” “Being furloughed,” “No employment”) at the time of the survey. Gender (female = 0, male = 1), marital status (single = 1), income change (furlough, decreased income = 1) were dummy coded. The reduced wage was coded together with being furloughed to capture the negative effects of COVID-19 on individuals' or familial financial capacities. In the 2020 data, provinces were coded according to the severity of exposure, with Hubei Province being 3, Guangdong and Zhejiang being 2, and Sichuan and Shanxi being 1.

Depression was measured with the 20-item CES-D scale (35), which captured an individual's level of depression and the frequency of thoughts or behaviors during the past week and used a three-point scale from 0 (< *1 day a week*) to 3 (*5–7 days a week*). The total score ranged from 0 to 60, with a higher rating indicating a more severe presentation of depression. Although Radloff's (35) original work supported a four-factor model of CES-D (i.e., depressed affect, positive affect, somatic and retarded activity, and interpersonal factor), the current investigation adopted a two-factor model to avoid potential over-extraction (36). The two factors were relevant to the wording of the items as four of them were positively valenced and the remaining negatively valenced (37). Factor ***positive emotion***included item 4, 8, 12, and 16, and the remaining items summed to reflect the second factor ***depressed*** ***mood***.

### Analytic Plan

All statistical analyses were performed in R (38) via glm in base R, and pequod, huge, car, tidyverse, lm.beta, lme4, WRS packages. To investigate the significance of differences in scores obtained from CES-D among the four groups (2016 and Mildly, Moderately, and Severely Impacted Areas in 2020) categorized by the levels of severity at which an area was hit by COVID-19, analysis of variance (ANOVA) or its variant would be applied after the assumptions of equal variance and normality were tested.

To explore the relationship between demographic factors and depression, depressed mood, and positive affect in 2020, three hierarchical multiple regression models were built. Standardized coefficients (β) were provided for regression analyses. Simple slope analysis (39, 40) was conducted on interaction effects to reveal the nature of significant interactions and detect relations between predictors and outcomes at different levels of the moderator with increased sensitivity (41). Compared to the test of interaction effect in a regression model, a test of simple slopes has increased power regardless of the interaction term's significance and decreased likelihood of Type II error, while maintaining an equivalent level of Type I error (41). Severity was set as the moderator for all analysis with each slope assessed at “low” (1 *SD* below the mean) and “high” (1 *SD* above the mean) levels of severity.

## Results

From the complete CFPS 2016 data set, a total of 950 respondents at their middle age (*M* = 43.33, SD = 13.57) were included for the calculation of norm and final analysis. The majority of respondents were women (54.8%), married, divorced, or widowed (87.1%), and attended high school education or less (80.4%).

For the 2020 sample, 1,200 participants at their middle age (*M* = 31.18, *SD* = 11.59) were eligible for final analyses. The sample size was moderate across Hubei (*N* = 300, Age: *M* = 29.26, *SD* = 9.99), Guangdong (*N* = 199, Age: *M* = 28.82, *SD* = 8.40), Zhejiang (*N* = 201, Age: *M* = 28.37, *SD* = 10.24), from Sichuan (*N* = 249, Age: *M* = 27.51, *SD* = 8.89), and Shanxi (*N* = 251, Age: *M* = 41.26, *SD* = 13.27). Among them, a majority were women (64.1%), single or never married (53.1%), had a master's degree or less (86.2%), an annual income of 50,000 CNY or less (48.2%), and did not report changes in their work or income status (23.6%).

### Change in Depression From 2016 to 2020

At the traditional CES-D cutoff value of 16 (35), which proposes that people who score equal to or above 16 are at risk for clinical depression, the relation between these variables was significant, *X*^2^ (1, *N* = 2,150) = 112.87, *p* < 0.001, and 29, 44, 49, and 54% residents met this criterion of depression in 2016, MiA, MoA, and SeA, respectively. Following the recommendations of Vilagut and colleagues (42) who proposed 20 as a better cutoff point, the relation was still significant, *X*^2^ (1, *N* = 2,150) = 97.95, *p* < 0.001, and 16, 32, 35, and 40% residents met this criterion of depression in 2016, MiA, MoA, and SeA, respectively.

**Figure 1:**
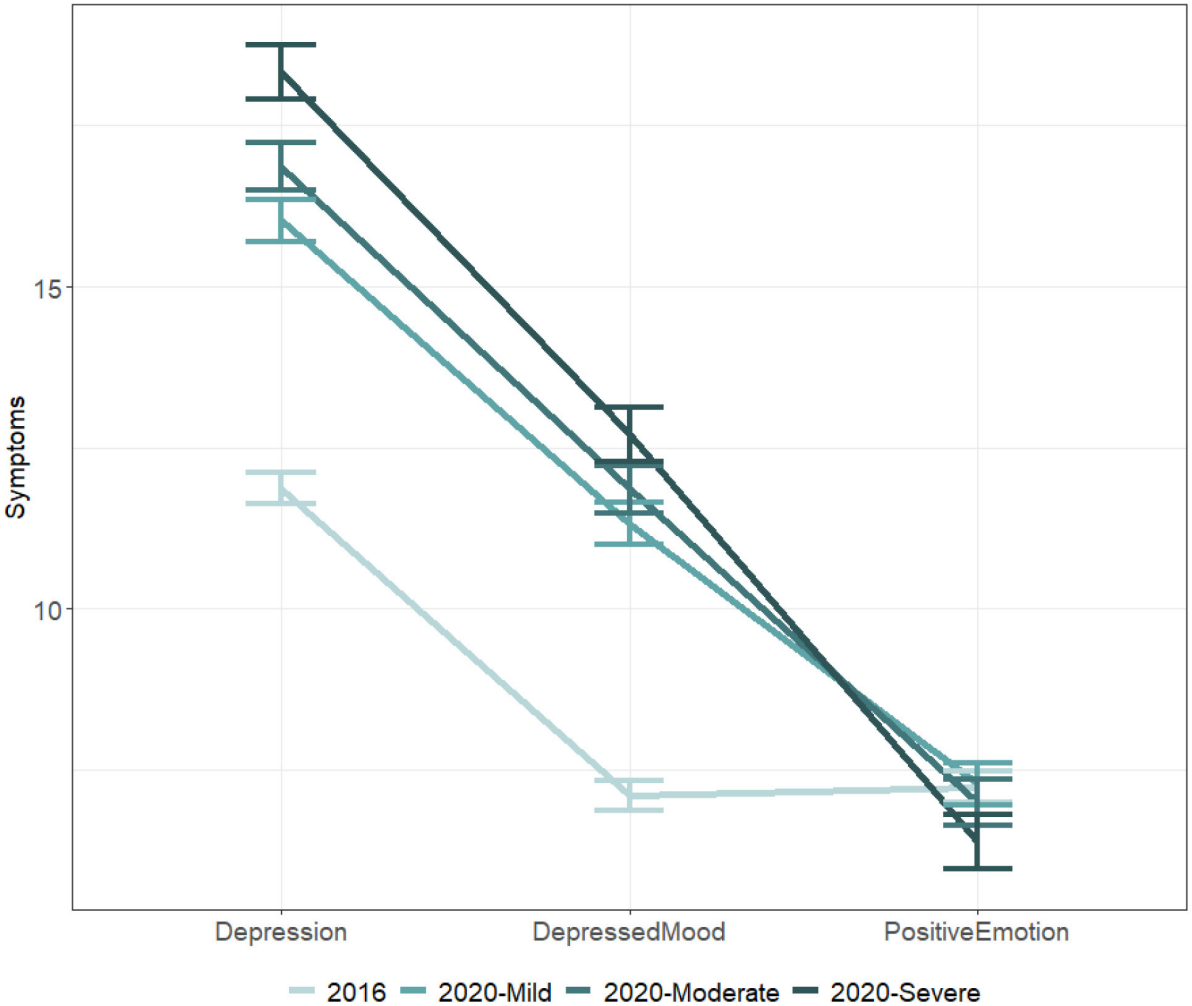
Comparison of depression, depressed mood, and positive emotion among residents in severely, moderately, and mildly impacted areas. *Note*. Error bars indicated standard errors.

As the data collected violated the assumptions (i.e., assumption of normality and equal variances) of traditional ANOVA, robust ANOVAs and robust *post-hoc* tests based on bootstrapping and trimmed means were chosen, as they could yield more accurate results when assumptions are not met (43). In total, three one-way robust ANOVAs were specified to evaluate the differences among four groups on total depression score, depressed mood, and positive emotion (Table 1 and Figure 1).

**Table 1:** Depression in Residents Assessed in 2016, MiA, MoA, and SeA and Symptom Comparisons.

**Symptoms**		**Depression**		**Depressed mood**		**Positive emotion**	
*M* (*SD*)	Range	[0 60]		[0 48]		[0 12]	
	2016	11.9 (7.82)		7.09 (6.37)		7.23 (2.78)	
	MiA	16.0 (11.1)		11.3 (9.37)		7.28 (3.12)	
	MoA	16.9 (10.3)		11.8 (8.58)		6.99 (2.99)	
	SeA	18.3 (10.4)		12.7 (8.85)		6.38 (2.77)	
		**Hedges'** ***g***	***p***	**Hedges'** ***g***	***p***	**Hedges'** ***g***	***p***
Symptom comparisons	2016<MiA	0.45	**<0.001**	0.56	**<0.001**	0.02	0.41
	2016<MoA	0.58	**<0.001**	0.66	**<0.001**	−0.08	0.17
	2016<SeA	0.75	**<0.001**	0.80	**<0.001**	−0.31	**<0.001**
	MiA<MoA	0.08	0.14	0.05	**<0.05**	−0.09	0.17
	MiA<SeA	0.21	**<0.01**	0.15	**<0.01**	−0.30	**<0.001**
	MoA<SeA	0.14	0.10	0.10	0.16	−0.21	**<0.01**

*(1) 2016 = Baseline Depression Norm (N = 950). MiA, Mildly Impacted Areas (N = 500); MoA, Moderately Impacted Areas (N = 400); SeA, Severely Impacted Area (N = 300). (2) Hedges' g values are considered large, medium, and small at 0.80, 0.50, and 0.20. (3) Bolded p-values are considered significant after robust post-hoc tests based on trimmed means and bootstrapping with false discovery rate set to be 0.05*.

A robust ANOVA examining possible differences among the four groups was significant, *Ft* = 45.15, *p* < 0.001, indicating that some groups reported elevated levels of depression than others. The results of robust *post-hoc* tests were all significant except for the comparisons between the scores of residents who lived in MoA and those of residents who lived in SeA, *p* = 0.10 and between MiA and MoA, *p* = 0.14. Notably, the robust *post-hoc* comparisons revealed that the residents in 2016 were significantly less depressed than those of residents living MiA, *p* < 0.001, Hedges' *g* = 0.45, MoA, *p* < 0.001, Hedges' *g* = 0.58, and SeA, *p* < 0.001, Hedges' *g* = 0.75, in 2020.

The second robust ANOVA revealed significant differences across groups in depressed mood, *Ft* = 61.65, *p* < 0.001. The results of robust *post-hoc* tests were almost always consistent with the previous *post-hoc* test on total depression: all results were significant except for the comparison between MoA and SeA, *p* = 0.16. Residents assessed in 2016 exhibited significantly lower levels of depressed mood than those in MiA, *p* < 0.001, Hedges' *g* = 0.56, MoA, *p* < 0.001, Hedges' *g* = 0.66, and SeA, *p* < 0.001, Hedges' *g* = 0.80, in 2020.

A final robust ANOVA on positive emotion also yielded a significant result, *Ft* = 7.39, *p* < 0.001, indicating that some groups experienced lower positive mood. The results of *post-hoc* tests, however, revealed a different pattern. Residents in SeA exhibited reduced positive mood than all other groups*, p.s*. < 0.01, Hedges' *g.s*. < −0.21. The level of positive emotions of residents in MoA and MiA in 2020 were not statistically different from those of residents assessed in 2016.

### Symptom Variability Within 2020

For the total score of ***depression***(Table 2), in step 1, age was found negatively associated with depression, β = −0.21, *t*_(1197)_ = −7.36, *p* < 0.001. In step 2, main effects of educational attainment, marital status, income level, and income change due to COVID-19 were included. As expected, a negative relationship between education and depression was found, β = −0.09, *t*_(1178)_ = −3.14, *p* = 0.002. Moreover, the experience of being furloughed was significantly associated with increased depression, β = 0.11, *t*_(1178)_ = 3.62, *p* < 0.001. Severity of COVID-19 was included to the model in step 3, but there was no evidence supporting the link between provincial severity of COVID and depression, β = 0.04, *t*_(1177)_ = 1.53, *p* = 0.13. In step 4, six two-way interaction terms, between gender and severity, age and severity, education and severity, being single and severity, income and severity, and being furloughed and severity were included. Among them, only the one between gender and severity was significant, β = −0.19, *t*_(1171)_ = −2.53, *p* = 0.01. Simple slope analyses were performed. Compared to men, women were more likely to experience depression when the severity was high, *B* = −2.67, *p* = 0.006, but not when the severity was low, *B* = −0.09, *p* = 0.92. The marginally significant interaction effect between education and severity, β = 0.57, *t*_(1171)_ = 1.95, *p* = 0.05, was also tested. The slope was significantly different than zero at a low level of severity, *B* = −0.90, *t*_(1171)_ = −3.53, *p* < 0.001, yet insignificant at a high level of severity, *B* = −0.15, *t*_(1171)_ = −0.60, *p* = 0.55, suggesting that only in provinces that were less severely impacted, as years of education increased, the total CES-D score decreased.

**Table 2:** Standardized regression coefficients and accounted variances for hierarchical multiple regression.

	**CESD - Total depression**				**CESD - Depressed mood**				**CESD - Positive emotion**			
	**Step 1**	**Step 2**	**Step 3**	**Step 4**	**Step 1**	**Step 2**	**Step 3**	**Step 4**	**Step 1**	**Step 2**	**Step 3**	**Step 4**
Age	−0.21^***^	−0.17^***^	−0.16^***^	−0.13	−0.22^***^	−0.20^***^	−0.20^***^	−0.15	0.07^*^	0.02	−0.01	0.02
Gender	−0.04	−0.04	0.04	0.14+	−0.06^*^	−0.06^*^	−0.06^*^	0.12	−0.03	−0.03	−0.04	−0.14+
Years of education (YoE)		−0.09^**^	−0.10^***^	−0.23^**^		−0.07^*^	−0.07^*^	−0.20^**^		0.12^***^	0.13^***^	0.25^**^
Single (dummy coded)		0.07	0.07	0.24^*^		0.05	0.05	0.20+		−0.09+	−0.10^*^	−0.25^*^
Income		−0.02	−0.03	0.01		−0.02	−0.02	0.01		0.03	0.04	−0.003
Furlough (dummy coded)		0.11^***^	0.10^***^	0.16^*^		0.11^***^	0.11^***^	0.16^*^		−0.05	−0.05	−0.10
Severity			0.04	−0.34			0.01	−0.30			−0.12^***^	0.32
Age × severity				−0.009				−0.04				−0.08
Gender × severity				−0.19^*^				−0.19^*^				0.10
YoE × severity				0.57+				0.51+				−0.52+
Single × severity				−0.18				−0.16				0.16
Income × severity				−0.04				−0.03				0.05
Furlough × severity				−0.06				−0.05				0.05
*R*^2^	0.047	0.07	0.072	0.083	0.057	0.075	0.075	0.085	0.006	0.03	0.044	0.052
Adjusted *R*^2^	0.046	0.065	0.067	0.073	0.056	0.07	0.07	0.075	0.004	0.025	0.039	0.041
Residual Std. error	10.464 (df = 1,197)	10.363 (df = 1,178)	10.357 (df = 1,177)	10.320 (df = 1,171)	8.738 (df = 1,197)	8.677 (df = 1,178)	8.680 (df = 1,177)	8.655 (df = 1,171)	3.003 (df = 1,197)	2.977 (df = 1,178)	2.955 (df = 1,177)	2.951 (df = 1,171)
*F*-statistic	29.776^***^ (df = 2; 1,197)	14.822^***^ (df = 6; 1,178)	13.055^***^ (df = 7; 1,177)	8.190^***^ (df = 13; 1,171)	36.279^***^ (df = 2; 1,197)	15.908^***^ (df = 6; 1,178)	13.647^***^ (df = 7; 1,177)	8.385^***^ (df = 13; 1,171)	3.421^**^ (df = 2; 1,197)	6.003^***^ (df = 6; 1,178)	7.780^***^ (df = 7; 1,177)	4.935^***^ (df = 13; 1,171)

Note: +, p < 0.10;

* , p < 0.05;

** , p < 0.01;

*** *, p < 0.001*.

Similar findings emerged for ***depressed mood***(Table 2). In step 1, both gender, β = −0.06, *t*_(1197)_ = −2.12, *p* = 0.03, and age, β = −0.22, *t*_(1197)_ = −7.95, *p* < 0.001, were significantly related to changes in depressed mood. In step 2, educational level, β = −0.07, *t*_(1178)_ = −2.39, *p* = 0.02, was negatively associated with depressed mood, while being furloughed, β = 0.11, *t*_(1178)_ = 3.76, *p* < 0.001, was positively associated with depressed mood. In step 3, severity was not related to changes in depressed mood, β = 0.01, *t*_(1177)_ = 0.39, *p* = 0.70. In step 4, only the interaction between gender and severity demonstrated a meaningful relationship to depressed mood, β = −0.19, *t*_(1171)_ = −2.57, *p* = 0.01, whereas the interaction between education level and severity was marginally significant, β = 0.51, *t*_(1171)_ = 1.72, *p* = 0.09. Therefore, simple slope analyses were performed to reveal the nature of interaction. Comparable to depression, females who lived in more severely impacted regions experienced increased depressed mood compared to males, *B* = −2.73, *t*_(1171)_ = −3.37, *p* < 0.001, but not when they lived in less severely impacted areas, *B* = −0.45, *t*_(1171)_ = −0.60, *p* = 0.55. Education, again, was a significant predictor of depressed mood only when severity was low, *B* = −0.60, *t*_(1171)_ = −2.77, *p* = 0.006, but not when severity was high, *B* = 0.008, *t*_(1171)_ = 0.04, *p* = 0.97.

Age was positively associated with ***positive emotions***(Table 2), β = 0.07, *t*_(1197)_ = 2.50, *p* = 0.01. In step 2, there was strong evidence that more years of education were associated with greater positive emotion, β = 0.12, *t*_(1178)_ = 3.99, *p* < 0.001. Neither income nor being furloughed was significant. In step 3, results demonstrated that, as severity increased, positive emotion decreased, β = −0.12, *t*_(1177)_ = −4.23, *p* < 0.001. In the last step, the final model supported the significance of education, single status, *p.s*. < 0.05, and the marginal significance of the interaction effect between educational attainment and severity, β = −0.52, *t*_(1171)_ = −1.76, *p* = 0.08. Well-educated individuals were less susceptible to a drop in positive emotion in both less and more severely affected regions, though the slope at low severity, *B* = 0.30, *t*_(1171)_ = 4.30, *p* < 0.001, was steeper and signified a greater power than the slope at high severity, *B* = 0.16, *t*_(1171)_ = 2.26, *p* = 0.02.

## Discussion

Amid the global outbreak of COVID-19, individuals may experience increased distress.

In accordance with the first hypothesis, participants recruited in the early May of 2020 reported greater severity of depression as compared with a pre-pandemic norm established using representative samples in 2016. It should be noted that there were age differences and that the simple manipulation of limiting the sample to an age range (i.e., 18–65) could not guarantee the equivalence of central measures between these group. Within in the 2020 sample, elevation of symptoms differed significantly from MiA, to SeA, determined by the provincial infection rates. Our results have provided confirming evidence for the second hypothesis as well as previous studies suggesting that increased exposure to COVID-19 through location, media, or infected cases predicted mental health problems (44). High levels of depression may also reflect comorbid anxiety, posttraumatic stress disorder, sleep disorder, suicidal ideation, domestic violence, substance use disorder as tested in other studies, and potentiate long-term consequences like cognitive impairments, psychosomatic symptoms, and behavioral changes (45).

Depressed mood, like the total level of depression, was higher in 2020 and positively associated with the severity of COVID-19. Findings from the robust ANOVA confirmed that people facing COVID-19 experienced a significantly higher degree of depressed mood, which was intensified by increased exposure to COVID-19, as symptoms were significantly lower in MiA than MoA and SeA.

A separate pattern emerged for positive emotion, which did not differ among the baseline norm, MiA, and MoA. Only residents of SeA had significant impairments in positive emotion when compared to the other three groups. Perceived discrimination may partly account for this observation, since according media only residents of Hubei (i.e., SeA) reported various forms of prejudice. Previous studies have established a relationship between perceived discrimination and alterations in affect, especially in stressful situations like the current one (46). One way to explain the distinct pattern emerged for positive emotion could be that the pandemic primarily exerted its negative impact through aggravating depressed mood without necessarily reducing positive affect.

### Demographic Correlates

#### Age and Marital Status

In the 2020 sample, people who were younger and single were affected more heavily by COVID-19 and more likely to have depression. People who were older reported lower levels of depressed mood and higher levels of positive emotions. Marital status was not associated with depressed mood or positive mood in our study.

In regard to age, Qiu et al. (23) showed that individuals between the ages of 18 and 30 or above 60 had the highest level of distress, recognizing that young people were more susceptible to stress-inducing information on social media and individuals older than 60 were more likely to feel threatened by the high mortality rate of COVID-19 among elderly. Our findings have partly confirmed their results, as younger individuals in the current sample were more likely to be depressed, have depressed mood, or to experience decreased positive emotions. First, young and single individuals had easy access to social media platforms, were more motivated to seek health, informational, and social support online, and more likely to get overwhelmed by the combination of accurate and faulty information (47). Second, they were more likely to quarantine alone and fared the worst with greater levels of future uncertainty in terms of academic and career progression (24). Third, higher levels of loneliness, financial distress, sleep problems, perceived stress and anxiety, and lower resilient coping were found among the younger population, compared to the older population (48). Furthermore, prior studies regarding SARS in 2003 have underscored the interplay between infection control measures, like wearing a mask and confinement within the home, to reinforce a sense of isolation (49) and subjective loneliness (50), which typically differed from an active lifestyle brimmed with vigor and gregariousness. A dramatic and enforced change in the frequency of social interactions and lonely feelings exerted detrimental effects on health, including impaired functionality, perceived decline in life quality and self-rated health (50).

#### Gender

Inconsistent with parts of the third hypothesis, gender was marginally significantly associated with depressed mood, but not with depression or positive emotion. Female participants reported higher levels of depressed mood as compared to their male counterparts. Our findings were partly consistent with relevant studies which proposed that females had a greater risk for depression, anxiety, and stress across nations during the pandemic, although these studies did not specify any subdimensions of depression (51–53). Gender differences in depression have been long established, looking at this issue through biological, psychological and social lenses (54). Under the unique circumstances of a global pandemic, the quarantine order might have led to forced and unwanted proximity with others, exposing women to escalated relationship difficulties or interpersonal problems. In extreme cases, rates of domestic violence grew as tensions built at home and victims were involuntarily confined with their abusers (55). It also pushed women to accept an overload of roles within the household and outside of it while adjusting to additional responsibilities (54).

#### Educational Attainment

Unlike other research in which the effect of education has been inconsistent (22–24), we validated parts of the third hypothesis concerning the effect of education and found that more years of education were promising in decreasing depression, depressed mood, and increasing positive emotions. Education could protect against both persistent sadness and anhedonia or diminished positive affect. Education may prove useful when dealing with stressful life events, thereby decreasing the likelihood of lifetime depression (56–58). Another potential explanation could be that individuals with lower levels of education were often subject to a furlough or a permanent layoff at times of an economic recession (59).

#### Employment and Income

The individual income level prior to the pandemic was not associated with depression, depressed mood, or positive emotions. Results contradicted our hypothesis and previous research (12). For example, Ettman et al. (59) saw a higher prevalence of financial stressors and probable depression in people with fewer assets, defined by household income, savings, house ownership, education, and being married. Another study reported that families with lower income levels had elevated symptoms of depression, anxiety, insomnia, and acute stress across various regions in China (60). On the contrary, high monthly income was found to be a risk factor for depression, anxiety, and sleep disorder by Wenning et al. (61). Discrepancies in conclusions may suggest a complex picture of the relationship between income, financial assets, and risk of depression, possibly mediated by geographical location, parental financial support, and culture-specific spending and saving practices. Geographical location determines a city's level of urbanization, the proportion of migrant workers, and living or rental housing unaffordability issues (62). Even though household income was generally higher in the first-tier cities, individuals, especially migrant workers, faced the reality of high rent stress (i.e., with a rent-to-income ratio going up to 50% in some cities), spatial inequality, and uncertainty due to short-term lease contracts (63). Parental financial support might be a potential confounder in our study, considering that we measured individual income per year without taking parental contribution into account. In light of the traditional values that held interdependence in high regard, Chinese parents are typically more determined to support their children financially until the children bear the role of a supporter (64). Moreover, with a high national savings rate of 59% in 2012 (65), the Chinese samples might have more savings in immediate possession, allowing even low-income individuals to endure the situation. These factors complicated the meaning of income and should be disentangled before unveiling the true nature of how income level associated with depression during the pandemic.

Partly supporting the third hypothesis, individuals who had reduced wages or were involuntarily furloughed from work faced a significantly higher chance of being depressed compared to individuals whose income or employment status was unruffled by COVID-19. More specifically, they were more vulnerable to elevated depressed mood but not diminished positive emotions. Being furloughed could be a burdensome financial stressor that also amplified feelings of uncertainty and helplessness, further exacerbating depression.

#### Severity

Severity was not directedly related to depression or depressed mood after controlling for several covariates. However, the severity of COVID-19 exposure was a moderator for the relationship between some demographic correlates and depression as well as depressed mood. First, female participants living in regions of high severity were most susceptible to depression and depressed mood when compared to male participants. Hence, in less severely impacted areas, like Shanxi or Sichuan, women and men were more equally affected by the repercussions of COVID-19 than their counterparts living in SeA, where females were at a disadvantage. Second, more years of education was related to decreased depression and depressed mood in mildly impacted areas, but not in severely impacted areas. Education, being a consistent protective factor against the destructive consequences of COVID-19, was more effective when risk remained low and manageable, yet as severity rose, education lost its benefits. The findings highlighted unique challenges that residents in SeA encountered, possibly due to a combination of stressors, including more significant perceived discrimination, stricter lockdown policy, and a higher risk of contracting the virus.

Contrary to the findings of depression and depressed mood, severity was negatively associated with changes in positive emotions. Neither of the six interaction terms yielded significant results. A subsequent simple slope analysis of the marginally significant relationship between education and severity suggested that more years of education was associated with greater positive emotion regardless of severity. However, the effect was stronger in MiA or MoA.

Gender, age, education, marital status, changes in employment status were pertinent factors to consider when studying changes in the psychological well-being of those facing the pandemic. Throughout the investigation, younger age and lower educational attainment were consistent risk factors for depression, whereas, gender, marital status, and being furloughed from work were situation-specific. The pattern of positive emotion was distinct from models of depression and depressed mood, suggesting that positive affect operated through a distinctive pathway to depression. The findings illustrated that outcomes differed depending on symptoms assessed, accentuating the need to identify symptoms of interest and to match them with the most appropriate and applicable scale or measurement approach.

### Limitation

Several limitations of the study should be acknowledged. The first limitation concerns the sampling strategy of the comparison norm. Our goals were to compare a sample collected during a normal situation (i.e., 2016) with one collected during a global emergency, and further examine whether there were differences among participants from severely, moderately, and mildly affected areas (i.e., 2020). Given the nature of a convenient sample in 2020, concerns might arise as to if they truly represented residents of each province investigated. Although we did adopt an adequate size of sample and recruited from multiple platforms to avoid sampling and estimation biases, future studies with both pre- and post-pandemic data in the same sample may better control for potential confounds. Second, the self-reported CES-D scale was only suitable for evaluating levels of depression and not anxiety, since they are highly comorbid, particularly given that 29% of respondents reported moderate to severe anxiety symptoms in another study (24). CES-D was adopted here because the primary interest was to assess depression. Other studies are encouraged to evaluate a wide range of outcomes. Third, actual stressors specific to COVID-19 and the experience of containment were not assessed and precluded to keep the survey brief. It is unclear how unique stressors, such as contact history with confirmed cases, health status, pre-existing mental disorder, lack of socioeconomic resources, and stigma, are related to psychological distress.

## Conclusion

The study examined a narrow range of psychological consequences of COVID-19 in Chinese residents who were living in MiA, MoA, and SeA, compared to a baseline group living in the same provinces in 2016. The outbreak was related to individuals' increased symptoms of depression, elevated levels of depressed mood, and diminished positive emotions. Stigma and local government policies may stir waves of distrust among neighbors and friends and feelings of marginalization and isolation, especially for residents in the Hubei province. Timely psychological interventions are necessary for individuals in need, particularly those who are single, unemployed due to COVID-19, and have fewer years of education.

## Data Availability Statement

The raw data supporting the conclusions of this article will be made available by the authors, without undue reservation.

## Ethics Statement

The studies involving human participants were reviewed and approved by Institutional Review Board of Tsinghua University, Department of Psychology. The patients/participants provided their written informed consent to participate in this study.

## Author Contributions

XH, ZY, KB, and SC designed the study and analyzed the data under the supervision of PS. XH, SC, KB, ZY, and PS discussed the results and wrote the paper together. All authors contributed to the article and approved the submitted version.

## Conflict of Interest

The authors declare that the research was conducted in the absence of any commercial or financial relationships that could be construed as a potential conflict of interest.
